# HbMYB44, a Rubber Tree MYB Transcription Factor With Versatile Functions in Modulating Multiple Phytohormone Signaling and Abiotic Stress Responses

**DOI:** 10.3389/fpls.2022.893896

**Published:** 2022-06-02

**Authors:** Bi Qin, Song-Le Fan, Hai-Yang Yu, Yan-Xi Lu, Li-Feng Wang

**Affiliations:** ^1^Key Laboratory of Biology and Genetic Resources of Rubber Tree, Ministry of Agriculture and Rural Affairs, Rubber Research Institute, Chinese Academy of Tropical Agricultural Sciences, Haikou, China; ^2^Institute of Tropical Crops, Hainan University, Haikou, China

**Keywords:** rubber tree, MYB transcription factor, HbMYB44, phytohormone signaling, stress tolerance

## Abstract

The vital roles of R2R3-MYB transcription factors (TFs) in regulating stress response and phytohormone signaling have been thoroughly studied in numerous plant species, but the functions of these TFs in rubber tree are poorly understood. Rubber tree is the most important source of natural rubber but often suffers from various abiotic and biotic stresses that cause severe yield losses each year. In this study, we reported a novel *MYB44* gene in rubber tree (named *HbMYB44*) and revealed its biological function. HbMYB44 was highly similar to AtMYB44 and clustered into subgroup 22. Transient expression indicated that HbMYB44 is a nuclear localized protein and displays transactivation activity at the C-terminus. *HbMYB44* was ubiquitously expressed in rubber tree, and its expression was strongly induced by multiple phytohormones, drought stress, wounding, and H_2_O_2_ treatments. Furthermore, overexpression of *HbMYB44* in *Arabidopsis* (OE) demonstrated that OE plants significantly enhanced stress tolerance, i.e., salt stress, osmotic stress, and drought stress. Additionally, *HbMYB44* promoted recovery from root growth inhibition of OE plants caused by exogenous phytohormones (including abscisic acid, methyl jasmonic acid, gibberellic acid 3, and salicylic acid), but the opposite effect was present in response to ethephon. Interestingly, *HbMYB44* increased the expression of its homologous genes and interacting protein-encoding genes in OE plants. Overall, *HbMYB44* plays versatile functions in modulating multiple phytohormone signaling pathways and stress tolerance.

## Introduction

The first MYB transcription factor (TF) was identified as a protein encoded by a viral oncogene (*v-MYB*) in birds (Wang X. et al., 2018). In plants, MYB TFs compose one of the most important families of TFs and regulate various plant development processes and multiple stress responses (Ampomah-Dwamena et al., 2019). MYB TFs can be classified into four groups according to their repeats (R) with 50–53 amino acid residues: 1R-, R2R3-, 3R-, and 4R-MYBs (Zhang T. et al., 2018). R2R3-MYB TFs play important roles in regulating plant development, abiotic and biotic stress responses, etc. For example, the kiwifruit *MYB7* modulates chlorophyll and carotenoid accumulation by activating the promoter of *lycopene beta-cyclase* (*AdLCY*-β) (Ampomah-Dwamena et al., 2019). Overexpression of wild soybean *GsMYB1* enhances resistance to salt stress and *Helicoverpa armigera* in transgenic *Arabidopsis* (Shen et al., 2018). Recently, advances in genome and transcriptome sequencing techniques have facilitated the identification of the MYB family in non-model plant species, such as peach (Zhang C. et al., 2018) and *Pleurotus ostreatus* (Wang L. et al., 2018).

The *Arabidopsis* R2R3-MYB family can divide into 25 subgroups based on the conserved motifs present in the C-terminus to the MYB domain. AtMYB44, together with AtMYB70, AtMYB73, and AtMYB77, belongs to subgroup 22, which contains two conserved motifs, namely, 22.1 (TGLYMSPxSP) and 22.2 (GxFMxVVQEMIxxEVRSYM) (Stracke et al., 2001). The MYBs of subgroup 22 play essential roles in regulating plant stress response and phytohormone signaling. For instance, *AtMYB73* expression was found to be upregulated under salt stress conditions in *Arabidopsis*, and it negatively regulated *SOS1* and *SOS3* transcripts (Kim et al., 2013). AtMYB44 confers abiotic stress tolerance and non-specific phytohormonal induction in *Arabidopsis* (Jung et al., 2008, 2010). Moreover, by activating *EIN2* expression, AtMYB44 functions in various biotic stress responses, such as resistance to *Myzus persicae* Sulzer and *Plutella xylostella* L. (Lu et al., 2013). On the one hand, AtMYB44 regulates salicylic acid (SA)- and jasmonic acid (JA)-mediated defense responses by directly regulating *WRKY70* expression; thus, this TF acts as an integrator of cross-talk between SA and JA in plant defense responses (Shim and Choi, 2013; Shim et al., 2013). On the other hand, AtMYB44 interacts with the abscisic acid (ABA) receptors PYL9 and PYL8 and regulates ABA signaling (Shim et al., 2013; Li et al., 2014; Zhao et al., 2014). Additionally, AtMYB44 is regulated by MPK3-mediated phosphorylation (Persak and Pitzschke, 2013). Recently, chromatin immunoprecipitation showed that several features present in the promoter region of *AtMYB44* facilitate the binding of different TFs involving various signals, including a low density of nucleosomes, no DNA methylation, and multiple TF-binding elements (Nguyen et al., 2012; Nguyen and Cheong, 2018a,b). Although the function of *MYB44* has been reported in the model plant species *Arabidopsis*, its function in non-model plant species is poorly understood. To improve our knowledge of its transcriptional regulation mechanism, the functions of *MYB44* in different species need to be characterized.

Rubber tree (*Hevea brasiliensis* Müll. Arg) is the most important plant species for the production of natural rubber (NR) (Puskas et al., 2006). Latex within NR is produced in the cytoplasm of laticifer cells and harvested by regularly tapping the bark of rubber trees. Ethephon (ET) is widely used in NR production to increase the latex yield. Rubber trees often suffer from abiotic stress, wounding (caused by frequent tapping), drought, low temperature, and biotic stress, especially the destructive disease South American leaf blight (SALB), which is still a serious threat to plantations. From a biological point of view, NR is important for disease and insect defense and high-temperature stress responses in rubber trees (Sharkey and Yeh, 2001). Although important roles of MYBs have been revealed in plants, few MYB TFs in rubber tree have been characterized. *HbMyb1* can suppress cell death induced by stress conditions in tobacco (Peng et al., 2011). The expression of *HbSM1* (a 1R-MYB) was shown to be induced by phytohormones and wounding (Qin et al., 2014). Recently, 44 *MYBs* (*HblMYB1*-*HblMYB44*) were identified from the laticifer transcriptome database and the genome database of rubber tree. Among them, HblMYB44 (belonging to a new group G3) and HblMYB19 (belonging to a new group G2) were shown to bind to the promoters of *HRT1*, *FDPS1*, and *SRPP* in yeast and increase their expression in transient expression assays of tobacco (Wang et al., 2017). Until now, the function of MYBs of subgroup 22 has remained unknown in rubber tree. Due to the important functions of MYB44 in the plant stress response and phytohormone signaling, we assumed that MYB44 homologs may play a vital role in the stress response in rubber tree. In this study, *HbMYB44* was cloned, and its function was characterized in rubber tree and transgenic *Arabidopsis*. These results will lay a good foundation for elucidating the transcriptional regulatory mechanism underlying the stress response in rubber tree.

## Materials and Methods

### Plant Materials and Treatments

The rubber tree clone CATAS7-33-97 was planted at the experimental farm of Danzhou, Hainan Province, China. At the flowering stage, samples of leaves, bark, latex, and flowers were collected from the same plants simultaneously. Tissue culture-generated seedlings were used for different stress and phytohormone treatments according to our reported method (Qin et al., 2015). Briefly, plants were subjected to drought stress treatment by continuously withholding water for 10 days, and the control plants were well-watered, with samples collected at the same time points. Wounds were made on the leaves by pinching with forceps, and untreated plants were used as controls. For chemical treatments, whole plants were sprayed with 2% (v/v) H_2_O_2_, 1.0% (v/v) ET, 200 mM methyl jasmonate (MeJA), 3 mM gibberellic acid 3 (GA_3_), 200 μM ABA, and 5 mM SA, respectively. All the chemicals were diluted in distilled water that included 0.05% (v/v) ethanol, and the control plants were sprayed with distilled water that included 0.05% (v/v) ethanol. Six plants were included in each treatment, and leaves from the six plants were harvested at each time point. Three different biological replicates were analyzed. The harvested samples were immediately frozen in liquid nitrogen and stored at −80°C until analysis.

### Cloning of *HbMYB44* and Determining Its Structural Features and Phylogenetic Analysis

The AtMYB44 (AT5G67300) sequence was subjected to tBLASTn searches of the transcriptome shotgun assembly (TSA) and genome database of *H. brasiliensis*. According to the obtained sequence, primers (MYB44-F1 and MYB44-R1, Table 1) were designed to amplify the full-length open reading frame (ORF) of *HbMYB44* from the rubber tree.

**TABLE 1:** Sequences of the primers used in this study.

Name	Sequence (5′ to 3′)	Name	Sequence (5′ to 3′)
MYB44-F1	GCCAAGCTTCCATTTTATGATTACT	MYB44-R1	CTCTGTACAATGCCCAAAATTATTC
MYB44-F2	TTAAAGAGATCGGTGAGTGC	MYB44-R2	CTGTACACATGCGAAGAAGA
MYB44-EcoRI-F1	CGGAATTCATGGCTGTTACTGGGAA	MYB44-BamHI-R1	CGGGATCCCTCAATCTTACTAATC
MYB44-▲C-EcoRI-F2	CGGAATTCTCTAATAACAACGACC	MYB44-BamHI-R2	CGGGATCCCTACTCAATCTTACTAA
MYB44-▲N-BamHI-R3	CGGGATCCAGACGACGTCGTTTCTA		
HbActin-F	GATGTGGATATCAGGAAGG	HbActin-R	CATACTGCTTGGAGCAAGA
AtMYB44-F	AAGCCCAACTGGATCTGATG	AtMYB44-R	GGTGGATCATCGGAAGAAGA
AtMYB70-F	GTAACAAGTGGGCGACGATT	AtMYB70-R	ACTCTGCCCTTCCTCTCCTC
AtMYB73-F	TGGGCTACGATCTCTCGTCT	AtMYB73-R	TCAACGGTTGCTCTTCTCCT
AtMYB77-F	GCGTTGATGTTTCCGAGATT	AtMYB77-R	TTTCCGCCATGTAACTCCTC
AtPYL8-F	CATCTCTCTTCACCCCGAGA	AtPYL8-R	ACCGCAAGACGTTCAGAGAT
AtPYL9-F	TACGGACGCATCATCAACAT	AtPYL9-R	ACTGCCGATTTCAGGATCAC
AtWRKY70-F	GAGGACGCATTTTCTTGGAG	AtWRKY70-R	GCTCAACCTTCTGGACTTGC
AtActin2-F	GATCTCCAAGGCCGAGTATGAT	AtActin2-R	CCCATTCATAAAACCCCAGC

*The restriction endonuclease site was underlined.*

The National Center for Biotechnology Information (NCBI) conserved domain searches^1^ and SMART^2^ were used to analyze the structure of HbMYB44. Sequences of *HblMYBs* identified from the laticifer transcriptome database and the genome database of *H. brasiliensis* (Wang et al., 2017), and *Arabidopsis MYBs* obtained from the phytozome,^3^ were used for the phylogenetic analysis. Information on *H. brasiliensis* and *Arabidopsis MYBs* is listed in Supplementary Table 1. Sequence alignments were conducted using the DNAMAN version 8.0 and ClustalX (Thompson et al., 2002). The phylogenetic analysis was performed using the MEGA version X based on the neighbor-joining method with a Poisson correction model and a bootstrap test of 1,000 replicates (Tamura et al., 2013).

### RNA Isolation and Analysis of Gene Expression

Total RNA was extracted from different tissues using a Total RNA Isolation kit (OMEGA Bio-tek, Norcross, GA, United States). cDNA was synthesized with ReverTra Ace qPCR RT Master Mix with gDNA Remover (TOYOBO, Shanghai, China) according to the manufacturer’s instructions. Quantitative real-time PCR (qRT-PCR) was performed on a CFX96™ Real-Time System (Bio-Rad Laboratories, Hercules, CA, United States) using the AceQ Universal SYBR qPCR Master Mix (Vazyme, Nanjing, China) according to the manufacturers’ instructions. Specific primers MYB44-F2 and MYB44-R2 were used for amplifying *HbMYB44* (Table 1). *HbActin* (GenBank accession number: HO004792) and *AtActin2* (GenBank accession number: NM_001338358.1) were used as housekeeping genes in the rubber tree and *Arabidopsis*, respectively. The relative expression levels of target genes were calculated using the 2^–ΔΔ*CT*^ method (Nolan et al., 2006; Schmittgen and Livak, 2008). All the data were analyzed by one-way ANOVA, and multiple comparisons were performed by the Tukey’s test at the *P* < 0.01 level. Figures were constructed by OriginPro 2018 (OriginLab Corporation, Northampton, MA, United States).

### Subcellular Localization of HbMYB44

To determine the subcellular localization of HbMYB44, a fluorescent GFP tag was fused to the C-terminus of HbMYB44. The ORF of *HbMYB44* was amplified with primers (MYB44-EcoR I-F1 and MYB44-BamH I-R1, Table 1) and subsequently inserted in the EcoR I/BamH I restriction sites of the vector 35S: XXGFP (provided by Dr. Xingliang Hou). The recombinant plasmid *35S:HbMYB44:mGFP* was then transformed into *Agrobacterium tumefaciens* strain GV3101, and then, the GV3101 strain harboring the recombinant plasmid was transiently expressed in tobacco (*Nicotiana benthamiana*) leaf epidermal cells according to the methods of our previous study (Qin et al., 2019). The fluorescent protein expression was examined by fluorescence imaging together with confocal microscopy (Leica, Wetzlar, Germany) after 1 day of dark/light cultivation post infiltration. The nuclei were stained with 4′,6-diamidino-2-phenylindole (DAPI) as previously reported (Kapuscinski, 1995) and visualized by confocal laser scanning microscopy.

### Transactivation Analysis in Yeast

The complete ORF of *HbMYB44* (amplified by MYBY44-EcoR I-F1 and MYBY44-BamH I-R2) and fragments of *HbMYB44* encoding the N-terminus [amino acids (aa) 1-200, amplified by MYBY44-EcoR I-F1 and MYBY44-▲N-BamH I-R3] or the C-terminus (aa 201-345, amplified by MYBY44-▲C-EcoR I-F2 and MYBY44-BamH I-R2), were amplified and cloned into the EcoR I/BamH II restriction sites of the vector pGBKT7,^4^ respectively. The target fragment was fused to the 3′ end of the GAL4 DNA-binding domain (DNA-BD) of the vector pGBKT7 between the EcoR I and BamH I restriction sites of MCS, such that a GAL4 DNA-BD-*HbMYB44* fusion protein would be produced when the yeast cells were transformed. The pGBKT7-HbMYB44, pGBKT7-▲N-HbMYB44, and pGBKT7-▲ C-HbMYB44 constructs; the negative control pGBKT7; and the positive control pGBKT7-HbNAC24 were used (HbNAC24 is an NAC TF of the rubber tree and has been shown to have transcriptional activation activity) (Kang et al., 2014). These constructs were individually transformed into yeast strain Y2H Gold using a Yeastmaker™ Yeast Transformation System 2 (Clontech, Mountain View, CA, United States) according to the manufacturer’s instructions. If the fusion protein had transactivation activity, the colonies containing the pGBKT7+fusion protein construct in Y2H Gold would grow on SD/-Trp/-His/-Ade (TDO) media and become blue on TDO supplemented with X-α-Gal (TDO/X) media. All the yeast transformants were grown at 30°C for 2–3 days on SD media lacking tryptophan (SD/-Trp). Then, the positive clones were transferred to TDO media or TDO/X media and cultured at 30°C for 2–3 days (Fujita et al., 2004; Zhang et al., 2014). The experiment was repeated three times.

### Plasmid Construction and Transformation of *Arabidopsis*

GV3101 cells harboring the recombinant plasmid 35S:HbMYB44:mGFP were used in *Arabidopsis* transformation for the overexpression of *HbMYB44* (OE). *Arabidopsis thaliana* Col-0 wild type (WT) was transformed *via* the floral-dip method as previously described (Guan et al., 2014). All the transgenic plants were selected on Murashige and Skoog (MS) media that included 10 mg L^–1^ Basta. Then, the resistant plants of the T2 generation were tested with the primers MYB44-EcoR I-F1 and MYB44-BamH I-R1 of *HbMYB44* and used for the following studies.

The expression of *HbMYB44*, its homologous genes (including *AtMYB44*, *AtMYB73*, *AtMYB70*, and *AtMYB77*), and genes (including *AtWRKY70*, *AtPYL8*, and *AtPYL9*) encoding the interacting proteins of AtMYB44 in OE and WT plants were analyzed using qRT-PCR according to the method mentioned above. The primers used are listed in Table 1.

### Phytohormone Response and Stress Tolerance Assays in Transgenic *Arabidopsis* Lines

The seeds of the T3 generation and WT seeds were sterilized with 10% bleach and 0.001% Triton X-100 for 10 min and washed five times with sterile water. The sterilized seeds were sown on MS media, stratified at 4°C in darkness for 2 days, and then transferred to a plant growth chamber (MMM/Climacell 707, Germany) set at 22°C with a 16 h light/8 h dark photoperiod (light intensity of 13,000 lux) for germination for 1–2 days. Then, the germinated seeds were transferred to MS media supplemented with 100 mM NaCl, 250 mM mannitol, 15% (w/v) PEG4000, 0.3 μM ET, 10 μM ABA, 20 μM MeJA, 50 μM GA_3_, and 10 μM SA. The plates were placed vertically in the illumination incubator under the same conditions used for germination, and the root length of the seedlings was measured after 10–13 days of treatments. For each treatment, at least six plants were tested in triplicate, and three different biological replicates were performed. The final value was the average of three batches of measurement data.

## Results

### HbMYB44 Is an R2R3-MYB Transcription Factor and Is Homologous to AtMYB44

**FIGURE 1:**
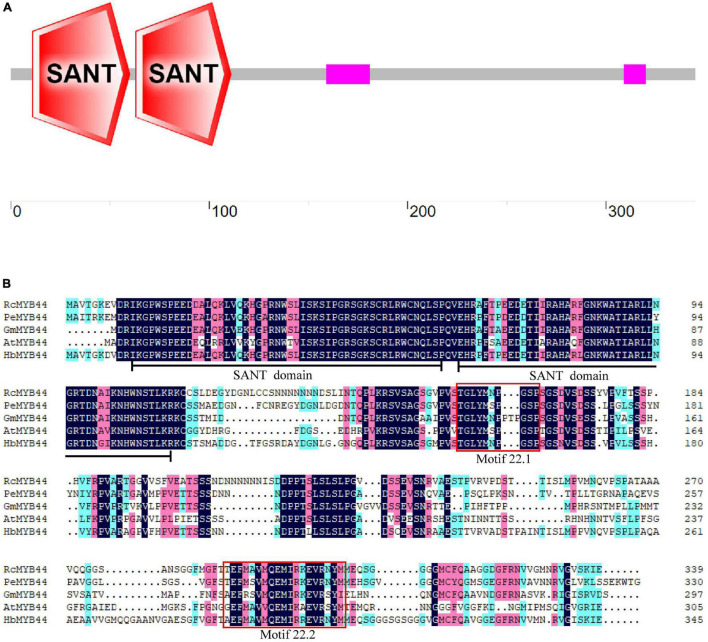
Analysis of HbMYB44 protein structure and multiple sequence alignment. **(A)** Protein architecture of HbMYB44. **(B)** Multiple sequence alignment of HbMYB44 and its homologous proteins. The proteins and their accession numbers are as follows, HbMYB44 (*Hevea brasiliensis*, JN038190), RcMYB44 (*Ricinus communis*, XP_002524926.1), PeMYB44 (*Populus euphratica*, XP_011006987.1), GmMYB44 (*Glycine max*, NP_001238087.1), and AtMYB44 (*Arabidopsis thaliana*, AT5G67300). Identical amino acids are marked with a dark background and well-conserved residues are marked with a pink background. Two SANT domains are underlined and two conserved motifs 22.1 and 22.2 are shown in red boxes.

The rubber tree *MYB44* homolog *HbMYB44* (GenBank accession number: AZZ09361.1) was cloned using RT-PCR. The ORF of *HbMYB44* is 1,038 bp in length and encodes a protein of 345 aa. No introns were found in the genomic sequence of *HbMYB44*. The deduced HbMYB44 protein contains 2 SANT domains (one is from the 11th to 60th aa, and the other is from the 63rd to 111th aa; Figures 1A,B), which indicated that HbMYB44 is an R2R3-MYB TF. A BLASTp search of the NCBI non-redundant (NR) protein database revealed that HbMYB44 was highly similar to MYB44 homologs from *Ricinus communis*, *Populus euphratica*, *Glycine max, and A. thaliana* with 72, 71, 61, and 55% identities, respectively (Figure 1B).

**FIGURE 2:**
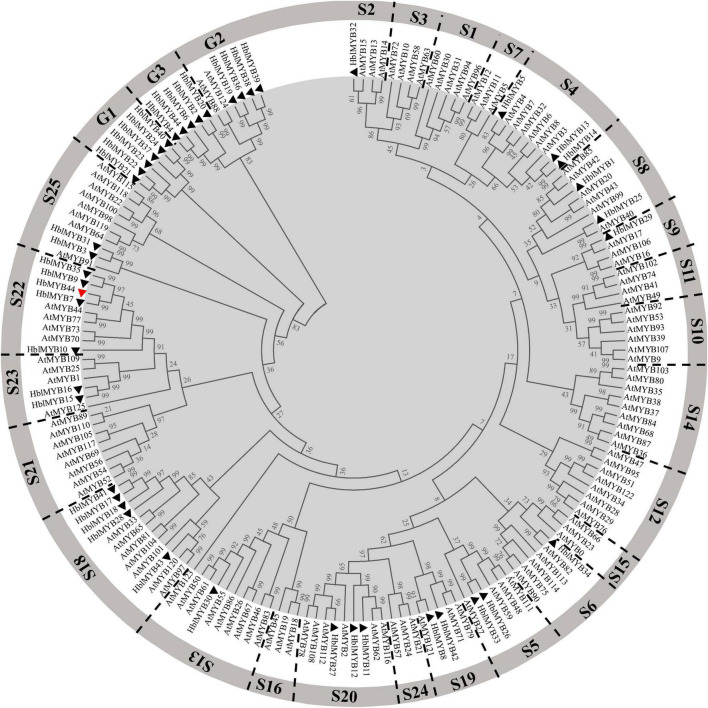
Phylogenetic tree analysis of HbMYB44 and the MYB family members of *Arabidopsis thaliana* and *Hevea brasiliensis. HblMYBs* identified from the laticifer transcriptome database and the genome database of *Hevea brasiliensis* (Wang et al., 2017) are indicated by triangles. Information on *Hevea brasiliensis* and *Arabidopsis* MYBs are listed in Supplementary Table 1.

Multiple sequence alignments between rubber tree and *A. thaliana* MYB family members indicated that HbMYB44 shared the highest similarity with rubber tree HblMYB7 (with 82% identity) and *Arabidopsis* AtMYB44 and AtMYB73 (with 55% identity). The phylogenetic analysis demonstrated that HbMYB44 clustered into subgroup 22 together with AtMYB44, AtMYB70, AtMYB73, AtMYB77, rubber tree HblMYB7, HblMYB9, HblMYB10, and HblMYB35 (Figure 2). Moreover, two specific motifs of subgroup 22, namely, motif 22.1 (TGLYMSPxSP) and motif 22.2 (GxFMxVVQEMIxxEVRSYM) were found to be present in HbMYB44 (Figure 1B, indicated by red boxes).

### HbMYB44 Is a Nuclear Protein With Transactivation Activity

**FIGURE 3:**
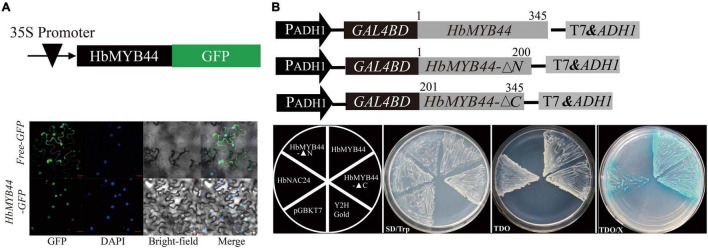
Subcellular localization and transactivation activity of HbMYB44. **(A)** Subcellular localization of HbMYB44 in the leaf epidermal cells of tobacco. Bars = 30 μm. **(B)** Analysis of the transactivation activity of HbMYB44 in yeast. The full length ORF of *HbMYB44*, and fragments encoding the N-terminus (1st–200th aa, *HbMYB44-▲N*) or the C-terminus (201st–345th aa, *HbMYB44-▲C*) of HbMYB44 were fused to the GAL4 DNA-binding domain of the pGBKT7 vector for the assay. TDO, SD/-Trp/-His/-Ade media; TDO/X, TDO media supplemented with X-α-Gal.

To determine the subcellular localization of HbMYB44, a fusion protein HbMYB44-GFP driven by the 35S promoter was generated and transformed into tobacco epidermal cells. The nucleus dyed with DAPI was blue, and HbMYB44-GFP fusion protein was green, both of which were cyan after being merged. The results demonstrated that HbMYB44-GFP was exclusively localized in the nucleus of tobacco epidermal cells, while the control empty vector free-GFP was distributed throughout the cells (Figure 3A). These results demonstrated that HbMYB44 is a nuclear protein.

Since MYB proteins function as TFs (Li et al., 2006), we tested the transactivation activity of HbMYB44. GAL4 DNA-BD-HbMYB44 fusion proteins were expressed in yeast, and their ability to activate transcription from the GAL4 upstream activation sequence and to promote yeast growth in defective media was analyzed. The full-length ORF of *HbMYB44* (HbMYB44), the N-terminal (encoding the 1st–200th aa, HbMYB44-▲N) and the C-terminal (encoding the 201st–345th aa, HbMYB44-▲C) fragments of *HbMYB44* were individually fused to the GAL4 DNA-BD within the pGBKT7 vector (Figure 3B) and transformed into yeast strain Y2H Gold for transactivation activity analysis as previously reported (Zhao T. et al., 2016). As shown in Figure 3B, all constructs (including pGBKT7-HbMYB44, pGBKT7-HbMYB44-▲N, pGBKT7-HbMYB44-▲C, negative control pGBKT7, and positive control pGBKT7-HbNAC24) grew on the SD/-Trp media, but only pGBKT7-HbNAC24, pGBKT7-HbMYB44, and pGBKT7-HbMYB44-▲C grew on the TDO media and appeared blue on TDO/X media. The full-length ORF of *HbMYB44* fused with the DNA-BD of GAL4 can self-activate the reporter genes in yeast cells. There was no effect on its transactivation activity when 200 aa within the N-terminus was deleted. However, truncation of 145 aa within the C-terminus resulted in the loss of transactivation activity. These results showed that HbMYB44 is a nuclear protein whose last 145 aa of the C-terminus have transactivation activity.

### *HbMYB44* Expression Was Induced in Response to Multiple Phytohormones and Stresses

**FIGURE 4:**
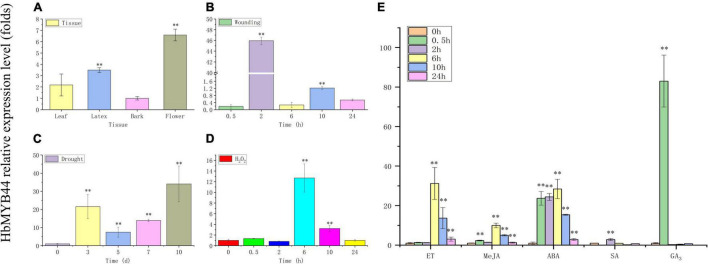
Expression of *HbMYB44* in different tissues and in response to stress and phytohormone treatments in rubber tree. The expression of *HbMYB44* was determined in different tissues of tapping trees **(A)** and in the leaves of tissue culture-generated seedlings subjected to wounding **(B)**, drought stress **(C)**, H_2_O_2_ **(D)**, and phytohormones **(E)**. The relative expression levels were compared with those at 0 h or 0 day. ^**^Indicates a significant differences at the *P* < 0.01 level.

The expression pattern of *HbMYB44* in rubber tree was measured using qRT-PCR. *HbMYB44* was expressed in all the tested tissues, but its expression in flowers was higher than that in other tissues; the expression in the flowers was nearly 6.5-fold higher than that in the bark (Figure 4A). Since MYBs of subgroup 22 play important roles in regulating the stress response and phytohormone signaling in plants, *HbMYB44* expression following different phytohormones and stress treatments was analyzed systematically. As shown in Figure 4B, *HbMYB44* expression was rapidly induced by wounding, and it was upregulated by 45.9-fold at 2 h after treatment. Under drought stress, *HbMYB44* expression was dramatically increased and peaked at 10 days after treatment, the level of which was 34.1-fold greater than that at 0 day (before treatment) (Figure 4C). After H_2_O_2_ treatment, *HbMYB44* expression was increased by 12.7-fold at 6 h (Figure 4D). Furthermore, *HbMYB44* expression was strongly induced in response to ET, MeJA, and ABA and peaked at 6 h, the levels of which were increased to 31.2-fold, 9.9-fold, and 28.4-fold, respectively (Figure 4E). In response to SA treatment, the *HbMYB44* expression level was upregulated by 2.8-fold at 2 h (Figure 4E). Interestingly, *HbMYB44* expression was rapidly triggered by GA_3_ application. *HbMYB44* expression peaked at 0.5 h, which was an increase of approximately 83-fold, but then quickly fell back at 2 h (Figure 4E). These results indicated that the *HbMYB44* expression was induced by multiple stress conditions and exogenous phytohormones.

### *HbMYB44* Overexpression Increases the Expression of Homologous Genes and Interacting Protein Genes in *Arabidopsis*

**FIGURE 5:**
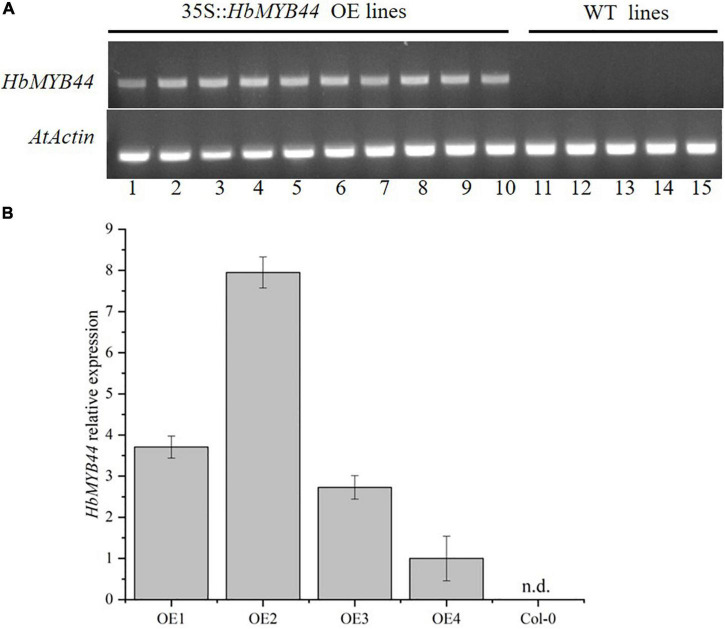
Verification of *HbMYB44* expression in transgenic *Arabidopsis.* **(A)** Validation of *HbMYB44* integration into the genome of transgenic plants. *Arabidopsis AtActin2* was used as a control. **(B)** Analysis of *HbMYB44* expression levels in transgenic plants by qRT-PCR. *AtActin2* was used as a housekeeping gene. Each value is the average of six plants tested in triplicate. n.d. means no detection.

To verify the function of *HbMYB44* in response to stress and phytohormones, transgenic *Arabidopsis* lines overexpressing *HbMYB44* (OE) were generated. The OE plants of the T2 generation were tested using PCR with the specific primers of *HbMYB44*, the results of which indicated that all 10 OE lines contained the target gene (Figure 5A). Then, the expression level of *HbMYB44* in OE lines was confirmed using qRT-PCR. There were differences in the expression abundance of *HbMYB44* among different transgenic lines, and OE2 with the highest expression was used for further analysis (Figure 5B).

**FIGURE 6:**
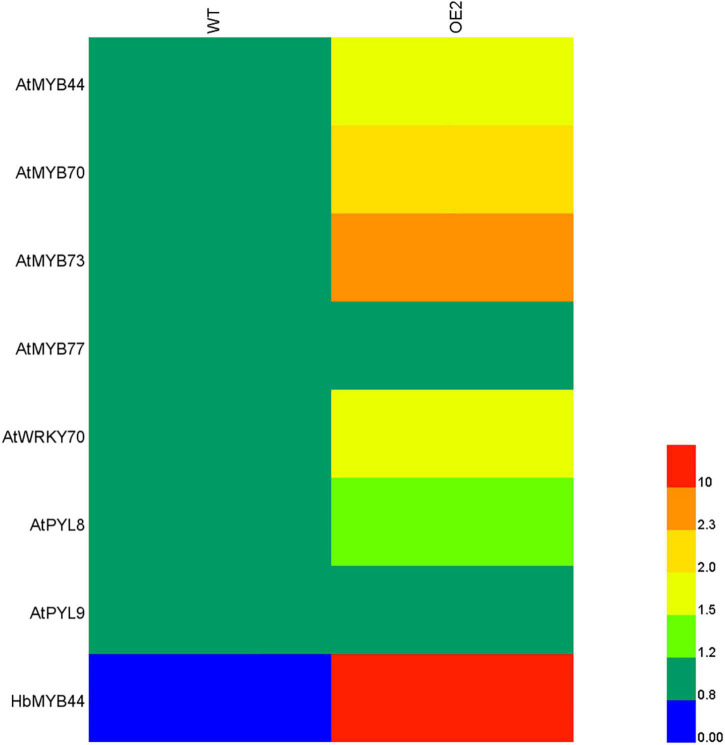
Expression of the subgroup S22 *MYB* and related genes in the *HbMYB44* overexpression line and WT. *AtActin2* was used as a housekeeping gene. Each value was the average of six plants tested in triplicate.

Since HbMYB44 functions as a TF with transactivation activity (Figure 3B), and its sequence is highly homologous to those of *Arabidopsis* MYBs of subgroup 22 (Figure 2), we analyzed whether HbMYB44 altered the expression of its homologous genes and their interacting proteins in transgenic *Arabidopsis* plants by qRT-PCR. As shown in Figure 6, except for that of *AtMYB77*, the expression of genes of subgroup S22, including *AtMYB44*, *AtMYB70*, and *AtMYB73*, was obviously increased in the *HbMYB44*-overexpressing plants compared with the WT plants. Moreover, the expression of *AtWRKY70* and *AtPYL8* but not *AtPYL9* was also obviously increased in OE plants. These results indicated that HbMYB44 enhanced the expression of its homologous genes and interacting protein-encoding genes in transgenic *Arabidopsis*.

### Overexpression of *HbMYB44* in *Arabidopsis* Enhances Tolerance to Abiotic Stress and Promotes Recovery From Root Growth Inhibition by Exogenous Phytohormones

**FIGURE 7:**
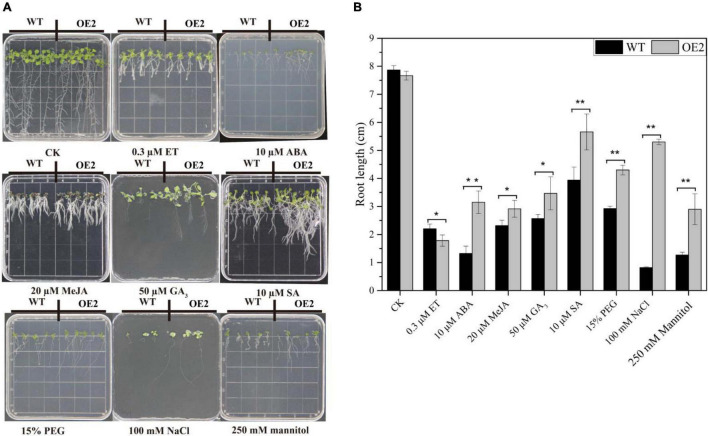
Phytohormone response and stress tolerance assays in the *HbMYB44* overexpression line and wild type (WT). **(A)** Phenotypes of the *HbMYB44* overexpression line and WT upon application of different phytohormones and stress conditions. **(B)** The root length of the seedlings under different phytohormone and stress treatments was measured after 10–13 days of treatments. Each value is the average of three batches of measurement data. The error bars represent ± SDs of three biological replicates, and the asterisks indicate significant differences between OE2 and WT plants (* and **Indicate *P* < 0.05 and *P* < 0.01, respectively).

Under normal conditions, there was no marked difference in the morphology of WT and OE plants cultivated in MS media, but different phenotypes, especially in terms of root growth, were found under stress and treatments involving exogenous phytohormones (Figure 7A). As shown in Figure 7, the growth of leaves and roots of both in WT and OE2 plants was severely suppressed under stress and exogenous phytohormone conditions. In the media supplemented with exogenous ABA, MeJA, GA_3_, and SA, the root length of OE plants significantly increased compared with that of the WT plants under the same conditions. However, the root length of OE plants was reduced compared with that of the WT plants in the media supplemented with ET (Figures 7A,B). These results demonstrated that the root growth of OE plants is less sensitive to ABA, MeJA, GA_3_, and SA, but more sensitive to ET. Under NaCl-salt stress, mannitol-mediated osmotic stress, and PEG-simulated drought stress conditions, the root lengths were markedly increased in the OE plants compared with the WT; the roots especially increased more than 5-fold under NaCl conditions (Figures 7A,B). These results indicated that *HbMYB44* enhanced tolerance to abiotic stress and promoted recovery from root growth inhibition in response to exogenous ABA, MeJA, GA3, and SA, suggesting that *HbMYB44* is involved in a complex phytohormone signaling network.

## Discussion

The DNA-BD of MYB TFs is essential for DNA recognition and transactivation (Ogata et al., 1996). However, significant differences in plant and animal MYB TFs are present in the domains of these TFs (Williams and Grotewold, 1997). Most plant MYB functions are related to their DNA-BD (Millard et al., 2019). The phylogenetic analysis showed that 125 R2R3-MYBs identified from the genome of *A. thaliana* can be divided into 25 subgroups (Stracke et al., 2001). There are two typical SANT domains present in subgroup 22 MYB TFs, enabling DNA-binding specificities and DNA interactions (Millard et al., 2019). Additionally, the subgroup 22 MYB TFs contain two conserved motifs, namely, motif 22.1 and motif 22.2, which are the binding domains of interacting proteins (Stracke et al., 2001; Li et al., 2014). This study showed that HbMYB44 is a member of subgroup 22, as supported by the phylogenetic analysis in which HbMYB44 is categorized into subgroup 22 together with AtMYB44, AtMYB70, AtMYB73, AtMYB77 and with rubber tree HblMYB7, HblMYB9, HblMYB10, and HblMYB35 (Figure 2). The two conserved motifs (22.1 and 22.2) are also present in HbMYB44 (Figure 1B). Moreover, HbMYB44 is located to the nucleus and has transactivation activity at the C-terminus. The region encompassing aa 201-345 is indispensable for transcriptional activation (Figure 3). These results indicated that HbMYB44 is a TF with transcriptional activation activity. Based on the conserved function of the genes in subgroup 22, it is speculated that HbMYB44, HblMYB7, HblMYB9, HblMYB10, and HblMYB35 in this subgroup may play an important role in phytohormone signaling and stress responses. Thus, further study should be carried out to verify the functions of these genes, which would help elucidate phytohormone signaling networks involved in plant defense and NR biosynthesis in rubber tree.

In plants, MYB44 is a key factor functioning as a bHLH-MYB-WRKY70 transcriptional module in plant non-specific phytohormone induction (Jung et al., 2010), defense responses (Shim and Choi, 2013; Shim et al., 2013), root elongation (Zhao Q. et al., 2016), stress resistance (Park et al., 2012), etc. Genes encoding the subgroup 22 MYB TFs have similar expression patterns and are associated with resistance to disease and stress. *AtMYB44* is expressed in different tissues throughout plants, with the highest abundance in the vasculature and stomata (Kirik et al., 1998). Moreover, *AtMYB44* expression was shown to be inducted in response to drought, cold, high salinity, CdCl_2_, sugars, wounding, SA, MeJA, ET, GA_3_, ABA, brassinosteroid, and cytokinin (Chen et al., 2006; Jung et al., 2008, 2010). The expression patterns of *AtMYB44*, *AtMYB73*, and *AtMYB77* were also shown to increase after wounding (Cheong et al., 2002), white-light (Ma et al., 2005) and cold stress treatments (Fowler and Thomashow, 2002). In this study, *HbMYB44* was expressed in all tissues, and it was strongly induced by multiple stresses (including wounding, drought stress, and H_2_O_2_ treatments) in rubber tree (Figure 4). Moreover, its transcript rapidly accumulated in response to phytohormones, and a significant increase in *HbMYB44* transcription was observed within 0.5 h after treatment with MeJA, ABA, and GA_3_ (Figure 4E). Therefore, *HbMYB44* transcripts accumulated non-specifically under diverse stress conditions and in response to various phytohormones, the results of which are similar to those of *AtMYB44* in *A. thaliana*, suggesting *HbMYB44* has functions similar to those of *AtMYB44*.

The stress response and growth regulation of MYB TFs are related to the phytohormone signaling pathway. For instance, AtMYB44 regulates *EIN2* expression, and *EIN2* is the key gene in ethylene signaling and insect resistance. Moreover, AtMYB44 regulates plant insect resistance by activating *EIN2* and its related defense responses in *Arabidopsis* (Lu et al., 2013). The ABA receptor RCAR3/PYL8 promotes lateral root growth by directly interacting with MYB77, MYB44, and MYB73, then enhancing MYB77-dependent transcription of auxin-responsive genes, to augment auxin signaling (Zhao et al., 2014). In the ABA signaling pathway, the ABA receptor RCAR1/PYL9 interacts with all the members of the subgroup 22 MYBs, i.e., AtMYB44, AtMYB70, AtMYB73, and AtMYB77 (Li et al., 2014). In *Solanum melongena*, the AtMYB44 homolog SmMYB44 enhances resistance to *Ralstonia solanacearum* by directly binding to the promoter of spermidine synthase (SPDS) gene *SmSPDS* and activating its expression (Qiu et al., 2019). These studies suggested that the members of subgroup 22 MYBs share similar functions and are functionally redundant. Through hormone crosstalk, AtMYB44 directly binds to the promoter of *AtWRKY70*, regulates *AtWRKY70* expression, and modulates antagonistic interactions between SA and JA signaling (Shim et al., 2013). Phytohormones play important roles in the NR production of rubber trees. The application of ET to the bark of rubber tree increases NR yield per tapping (Kush, 1994), and JA treatment of the bark can promote secondary laticifer differentiation and subsequent NR biosynthesis (Haoji-Lin, 2000; Deng et al., 2018). Rubber tree plantations often suffer from various biotic stresses and abiotic stresses, especially wounding (caused by tapping in the bark for NR harvesting). Thus, the identification and functional characterization of stress response and phytohormone signaling genes are of great significance for improving plant stress resistance and NR yield in rubber trees. In this study, overexpression of *HbMYB44* in *Arabidopsis* demonstrated that OE plants enhanced resistance to mannitol-mediated osmotic stress, NaCl-salt stress, and PEG-simulated drought stress (Figure 7). Moreover, *HbMYB44* promoted recovery from root growth inhibition caused by ABA, MeJA, GA_3_, and SA (Figure 7). In addition, the leaf biomass of OE plants was higher than those of the WT plants in the media supplemented with GA_3_, and the root biomass of OE plants was higher than those of WT plants in the media supplemented with SA (Figure 7A). These results demonstrated that *HbMYB44* is involved in a complex phytohormone signaling network. Interestingly, expression of the genes of subgroup S22 (including *AtMYB44*, *AtMYB70*, and *AtMYB73*) and interacting protein-encoding genes (*AtWRKY70* and *AtPYL8*) were significantly increased in OE plants (Figure 6). These results suggested that *HbMYB44* enhances stress tolerance and modulates phytohormone induction by increasing the expression of its homologous genes and interacting protein-encoding genes in *Arabidopsis*. Therefore, overexpression of *HbMYB44* in the rubber tree will be further conducted to determine its function in NR biosynthesis, stress resistance, and phytohormone signaling.

## Conclusion

The rubber tree HbMYB44 is a member of the subgroup 22 MYB TFs, which functions in the nucleus and has transactivation activity *via* its C-terminus. *HbMYB44* expression was strongly induced in response to multiple phytohormones and stresses. The overexpression of *HbMYB44* in *Arabidopsis* enhanced tolerance to abiotic stress and promoted recovery from root growth inhibition by exogenous phytohormones. This study suggested that *HbMYB44* plays an important role in modulating multiple phytohormone signaling pathways and stress resistance of rubber tree.

## Data Availability Statement

The original contributions presented in the study are included in the article/Supplementary Material, further inquiries can be directed to the corresponding author.

## Author Contributions

BQ was the experimental designer and the executor of the experimental research, completed the data analysis, and wrote the first draft of the manuscript. BQ, S-LF, H-YY, and Y-XL participated in experimental design, performed experimental research, and experimental result analysis. BQ and L-FW directed the design of the experiment, data analysis, and the modification of the manuscript. All authors read and approved the final manuscript.

## Conflict of Interest

The authors declare that the research was conducted in the absence of any commercial or financial relationships that could be construed as a potential conflict of interest.

## Publisher’s Note

All claims expressed in this article are solely those of the authors and do not necessarily represent those of their affiliated organizations, or those of the publisher, the editors and the reviewers. Any product that may be evaluated in this article, or claim that may be made by its manufacturer, is not guaranteed or endorsed by the publisher.

## Footnotes

1
http://www.ncbi.nlm.nih.gov/Structure/cdd/wrpsb.cgi?

2
http://smart.embl-heidelberg.de/

3
https://phytozome.jgi.doe.gov/pz/portal.html

4
www.clontech.com

## Abbreviations

aa: amino acids
ABA: abscisic acid
DAPI: 4’,6-diamidino-2-phenylindole
DNA-BD: DNA-binding domain
ET: ethephon
GA_3_: gibberellic acid 3
GFP: green fluorescent protein
MeJA: methyl jasmonic acid
NR: natural rubber
PEG: polyethylene glycol
SA: salicylic acid
TDO: SD/-Trp/-His/-Ade
TF: transcription factor
TSA: transcriptome shotgun assembly
X- α-Gal: 5-bromo-4-chloro-3-indolyl- α-d -galactopyranoside.
